# Bounding measures of genetic similarity and diversity using majorization

**DOI:** 10.1007/s00285-018-1226-x

**Published:** 2018-03-22

**Authors:** Alan J. Aw, Noah A. Rosenberg

**Affiliations:** 1 Mathematical and Computational Science Program, Stanford University, Stanford, CA 94305 USA; 2 Department of Biology, Stanford University, Stanford, CA 94305 USA

**Keywords:** Genetic diversity, Homozygosity, Majorization, Shannon and Rényi entropies, 26B25, 26D15, 92D10

## Abstract

The homozygosity and the frequency of the most frequent allele at a polymorphic genetic locus have a close mathematical relationship, so that each quantity places a tight constraint on the other. We use the theory of majorization to provide a simplified derivation of the bounds on homozygosity *J* in terms of the frequency *M* of the most frequent allele. The method not only enables simpler derivations of known bounds on *J* in terms of *M*, it also produces analogous bounds on entropy statistics for genetic diversity and on homozygosity-like statistics that range in their emphasis on the most frequent allele in relation to other alleles. We illustrate the constraints on the statistics using data from human populations. The approach suggests the potential of the majorization method as a tool for deriving inequalities that characterize mathematical relationships between statistics in population genetics.

## Introduction

Population-genetic summary statistics—functions computed from data on genetic variation—provide a central tool in population-genetic data analysis. Summary measures of allele frequencies, including measures of genetic similarity and diversity, are often computed from data, leading to much subsequent interpretation and additional computation.

Recent studies have demonstrated that pairs of population-genetic summary statistics often have a close mathematical relationship, so that values of one quantity strongly constrain the values of a second quantity (Hedrick 1999, 2005; Long and Kittles 2003; Rosenberg and Jakobsson 2008; Maruki et al. 2012; Jakobsson et al. 2013; Edge and Rosenberg 2014). For example, for a polymorphic locus, Rosenberg and Jakobsson (2008) showed that two measures of the homogeneity of the allele frequencies in a population—homozygosity and the frequency of the most frequent allele—tightly constrain each other over the unit interval, so that each can be predicted by the other to within 1 / 4, and so that the mean range over the unit interval for one of the statistics given the other is $\frac{2}{3}-\frac{\pi ^2}{18}\approx 0.1184$. Reddy and Rosenberg (2012) then tightened the bounds placed by homozygosity on the frequency of the most frequent allele and vice versa, in the case of a fixed value for the number of distinct alleles. These theoretical results provide guidance for interpreting computations of homozygosity and the frequency of the most frequent allele, including in homozygosity-based tests for evidence of natural selection (Rosenberg and Jakobsson 2008; Garud and Rosenberg 2015).

To further advance the study of mathematical properties of population-genetic statistics describing similarity and diversity of alleles in a population, we investigate the application of the theory of majorization—a mathematical and statistical approach concerned with evenness of arrayed structures (Marshall et al. 2010)—to these statistics. Majorization provides general principles concerning maxima and minima, enabling bounds to be obtained for broad classes of functions. In addition, as a theory about mathematical functions in general, it readily suggests new functions to be used as summary statistics, functions whose mathematical bounds are achieved at the same allele frequency distributions that generate bounds for existing statistics. We exploit this aspect of majorization to investigate the relationship of the frequency of the most frequent allele to various homozygosity-related statistics as well as to the Shannon–Weaver entropy statistic for genetic diversity.

First, we formally introduce the population-genetic statistics of interest. Next, we describe the majorization framework and establish its relationship with convex functions, the key connection that enables the derivation of our mathematical bounds. We then obtain bounds given a fixed size for the frequency of the most frequent allele on homozygosity statistics and the Shannon–Weaver index. We provide simpler derivations of results reported by Rosenberg and Jakobsson (2008) and Reddy and Rosenberg (2012); these derivations naturally lead us to consider a larger family of homozygosity-related statistics that we call $\alpha$-*homozygosities*, whose extreme values occur at the same allele frequency vectors as for standard homozygosity. Similarly, the Shannon–Weaver bounds can be extended to provide bounds on the more general Rényi entropies. We compare the constraints placed by the frequency of the most frequent allele on $\alpha$-homozygosity for various choices of $\alpha$, by applying the bounds to data on 783 multiallelic microsatellite loci in a sample of 1048 individuals drawn from worldwide human populations. The comparison helps extend the understanding of the empirical relationships among statistics describing genetic similarity and diversity in populations.

## Preliminaries

### Statistics based on allele frequencies

Consider a population and a polymorphic genetic locus with *I* distinct alleles. We represent the allele frequencies of the locus by the frequency vector $\mathbf {p}=(p_1,\ldots ,p_I)$. The components of $\mathbf {p}$ are arranged in descending order such that $p_i\geqslant p_j$ if $i<j$. We sometimes denote $p_1$, the largest allele frequency, by *M*. For all *i*, $p_i\in [0,1]$, and the entries in $\mathbf {p}$ sum to 1. Note that $p_1>0$.

Homozygosity for a locus is the sum of the squares of allele frequencies at the locus,

$$\begin{aligned} J=J(\mathbf {p})=\sum _{i=1}^Ip_i^2. \end{aligned}$$

For diploid loci at Hardy–Weinberg proportions, *J* measures the total frequency of homozygotes in a population. For loci of any ploidy, it provides a measure of the homogeneity of an allele frequency distribution, approaching 0 for a distribution consisting of many rare alleles and equaling 1 if a distribution has only a single allelic type.

We also consider a generalization that we term $\alpha$-homozygosity, which we define by

$$\begin{aligned} J^\alpha =J^\alpha (\mathbf {p}) =\sum _{i=1}^Ip_i^\alpha , \end{aligned}$$

where $\alpha >1$. The standard homozygosity *J* is the $\alpha$-homozygosity for the case of $\alpha =2$.

Next, we examine the Shannon–Weaver entropy index of diversity, also known as the Shannon–Wiener or Shannon index. This quantity is defined by

$$\begin{aligned} H=H(\mathbf {p})=-\sum _{i=1}^I p_i\log p_i, \end{aligned}$$

taking $0\log 0=0$. Unlike homozygosity, this index increases with diversity in the allele frequency distribution, rather than with homogeneity.

Note that the Shannon–Weaver index is a limiting case of the Rényi entropies, a family of diversity measures indexed by a variable $\alpha$. For a specified value of $\alpha \in (0,1)\cup (1,\infty )$, the Rényi entropy of order $\alpha$, or $\alpha$-entropy for short, is defined by

2.1 $$\begin{aligned} H^\alpha (\mathbf {p}) = \frac{1}{1-\alpha }\log \left( \sum _{i=1}^I p_i^\alpha \right) . \end{aligned}$$

$H^\alpha$ and $J^\alpha$ are related by $H^\alpha =\frac{1}{1-\alpha }\log (J^\alpha )$. For $\alpha = 1$, by l’Hôpital’s rule, $\lim _{\alpha \rightarrow 1} H^\alpha (\mathbf {p}) = H(\mathbf {p})$ for any allele frequency vector $\mathbf {p}$. We can then identify $\lim _{\alpha \rightarrow 1} H^\alpha$ with the Shannon–Weaver index *H*, treating the Shannon–Weaver index as the $\alpha = 1$ case of the Rényi entropy.

### Majorization

The theory of majorization (Marshall et al. 2010) is concerned with the notion of evenness in comparisons between pairs of vectors. Given a vector $\mathbf {v}=(v_1,\ldots ,v_I)$ with *I* components, let $v_{[i]}$ denote its *i*th largest component, which is not necessarily the same as its *i*th component, $v_i$. For example, if $\mathbf {v}=(1,5,3,4)$, then $v_{[1]}=5$ but $v_1=1$, and $v_{[2]}=4$ but $v_2=5$. For a pair of vectors $\mathbf {v},\mathbf {w}$ whose elements have the same sum, we compare their evenness by saying that $\mathbf {v}$ *majorizes* $\mathbf {w}$ if $\mathbf {w}$ is “more evenly distributed,” or “less concentrated,” than $\mathbf {v}$. The following definition formalizes this concept.

#### Definition 2.2

(*Majorization*) Let $\mathbf {v},\mathbf {w}\in \mathbb {R}^I$. Then $\mathbf {v}$ *majorizes* $\mathbf {w}$ if both of the following conditions hold.

1. $$\sum _{i=1}^Iv_i=\sum _{i=1}^Iw_i$$.
2. For each *k* from 1 to *I*, $\sum _{i=1}^k v_{[i]}\geqslant \sum _{i=1}^k w_{[i]}$.

If $\mathbf {v}$ majorizes $\mathbf {w}$, then we write $\mathbf {v}\succ \mathbf {w}$.

The first condition states that the vectors have the same sum of components. The second condition states that for each *k*, the sum of the *k* largest components of $\mathbf {v}$ is greater than or equal to the corresponding sum of the *k* largest components of $\mathbf {w}$. It can be verified from the definition that (0.75, 0.25) majorizes (0.5, 0.5) and (0.8, 0.1, 0.05, 0.05) majorizes (0.4, 0.3, 0.15, 0.15), but neither (0.5, 0.25, 0.25) nor (0.4, 0.4, 0.2) majorizes the other. Note that if $\mathbf {v}\succ \mathbf {w}$ and $\mathbf {w}\succ \mathbf {v}$, then $\mathbf {w}$ must be a permutation of $\mathbf {v}$. This result follows from the fact that if $\mathbf {v}\succ \mathbf {w}$ and $\mathbf {w}\succ \mathbf {v}$, then $\sum _{i=1}^k v_{[i]} = \sum _{i=1}^k w_{[i]}$ for each *k* from 1 to *I*.

In the $(I-1)$-dimensional simplex $\Delta _{I-1}=\{(p_i)_{i=1}^I:p_i\geqslant 0, \sum _{i=1}^Ip_i=1\}$ consisting of nonnegative vectors that sum to 1, $(1,0,\ldots ,0)\succ (1/2,1/2,0,\ldots ,0)\succ \cdots \succ (1/I,\ldots ,1/I)$. Moreover, for any $\mathbf {p}\in \Delta _{I-1}$, $(1,0,\ldots ,0)\succ \mathbf {p}\succ (1/I,\ldots ,1/I)$.

### Functions preserving majorization

Because majorization ranks vectors by evenness, it is natural to identify mathematical functions that preserve the ranking order. Such functions are termed *isotone* with respect to majorization: if $\mathbf {v}$ majorizes $\mathbf {w}$, then an isotone function *F* outputs a value $F(\mathbf {v})$ at least as large as $F(\mathbf {w})$. A function is termed *antitone* with respect to majorization, if whenever vector $\mathbf {v} \succ \mathbf {w}$, the function outputs a smaller or equal value. An isotone function is largest at maximal vectors with respect to the majorization order and smallest at minimal vectors, whereas the reverse is true for an antitone function.

An isotone function with respect to majorization provides a sensible index of concentration of vectors, whereas an antitone function provides a sensible index of diversity. As we shall see below, homozygosity is isotone, whereas the Shannon–Weaver index is antitone.

The functions that are isotone, or preserve the majorization order, are closely related to convex functions. Suppose *S* is a set of *I*-dimensional vectors, possibly $\mathbb {R}^I$. A function $F:S\rightarrow \mathbb {R}$ that preserves majorization on *S* is termed *Schur-convex*; the Schur-convex functions are by definition the isotone functions. Mathematically, *F* is Schur-convex if $\mathbf {v}\succ \mathbf {w}$ on *S* implies $F(\mathbf {v})\geqslant F(\mathbf {w})$. Furthermore, *F* is *strictly* Schur-convex if $F(\mathbf {v})> F(\mathbf {w})$ whenever $\mathbf {v}\succ \mathbf {w}$ but $\mathbf {v}$ is not a permutation of $\mathbf {w}$. A function *F* is *Schur-concave* if $-F$ is Schur-convex; Schur-concave functions are the antitone functions.

A useful method for identifying Schur-convex functions is the *Schur–Ostrowski criterion* (Marshall et al. 2010 pg. 84).

#### Theorem 2.3

(Schur–Ostrowski criterion) If *F* is symmetric in the components of its argument and all its first partial derivatives exist, then *F* is Schur-convex if and only if for every $\mathbf {v}\in S$,

$$\begin{aligned} (v_i-v_j)\left( \frac{\partial F}{\partial v_i}-\frac{\partial F}{\partial v_j}\right) \geqslant 0 \end{aligned}$$

for each pair of components of $\mathbf {v}$, $v_i$ and $v_j$. Moreover, *F* is strictly Schur-convex if equality requires $v_i = v_j$. The inequality is reversed for Schur-concave *F*.

We will have occasion to use a particular case of the Schur–Ostrowski criterion. The first derivative of a differentiable convex function $f:\mathbb {R}\rightarrow \mathbb {R}$ of one variable is by definition increasing. Therefore, for any pair of points $v_i,v_j$ at which *f* is differentiable, $f'(v_i)-f'(v_j)$ and $v_i-v_j$ have the same sign, implying that $(v_i-v_j)[f'(v_i)-f'(v_j)]\geqslant 0$. Hence, a function *F* that is decomposable into individual, identical convex functions in each of its arguments satisfies the Schur–Ostrowski criterion. An analogous statement holds for Schur-concave functions in relation to concave functions. We state this claim formally.

#### Corollary 2.4

If $\mathbf {v}=(v_1,\ldots ,v_I)$ and $F(\mathbf {v})=f(v_1)+\cdots +f(v_I)$ where *f* is (strictly) convex, then *F* is (strictly) Schur-convex. Similarly, if *f* is (strictly) concave, then *F* is (strictly) Schur-concave.

This corollary, in which the part about strict convexity appears on p. 92 of Marshall et al. (2010), is conveniently stated in the following form (Karamata 1932; see also pp. 156–157 of Marshall et al. 2010).

#### Theorem 2.5

(Karamata’s inequality) Let $S\subseteq \mathbb {R}$ be an interval and let $f:S\rightarrow \mathbb {R}$ be convex, and let $v_1,\ldots ,v_I,w_1,\ldots ,w_I\in S$. If $\mathbf {v} \succ \mathbf {w}$, then

$$\begin{aligned} f(v_1)+\cdots +f(v_I)\geqslant f(w_1)+\cdots +f(w_I). \end{aligned}$$

If *f* is concave, then the inequality is reversed. If *f* is strictly convex or strictly concave, then equality holds if and only if the list of values $w_1, \ldots , w_I$ gives a permutation of $v_1, \ldots , v_I$.

With these tools, we are now ready to study the constraints placed on *J* and *H* by *M*. In the next section, we apply Karamata’s inequality to establish bounds on these statistics as functions of the fixed largest allele frequency. In establishing Theorems 3.2 and 3.9, the main results from which we obtain the bounds on the statistics, the proofs follow a similar general structure. In each proof, we characterize the vectors that lie at extremes with respect to the majorization order among the vectors in a space. We then show that for functions on that space satisfying convexity conditions, extreme values occur at vectors that are extreme with respect to the majorization order.

## Results

We consider bounds on population-genetic statistics in terms of the frequency *M* of the most frequent allele in two settings. First, we consider an unspecified number of distinct alleles (Sect. 3.1). This section includes the bounds of Rosenberg and Jakobsson (2008) and our new bounds on $\alpha$-homozygosity. Second, we consider a specified number of distinct alleles (Sect. 3.2). This section includes the bounds of Reddy and Rosenberg (2012) and new bounds on $\alpha$-homozygosity, the Shannon–Weaver index, and the Rényi entropy.

To obtain the bounds, we rely on the observation that $f(x)=x^\alpha$ is strictly convex for $\alpha >1$ and $x\geqslant 0$, so that $J^\alpha$ is strictly Schur-convex by Corollary 2.4. The convexity of $f(x)=x^\alpha$ follows from the second derivative test for convexity, by which *f* is convex if $f''(x)\geqslant 0$, as $f''(x)=\alpha (\alpha -1)x^{\alpha -2}>0$ for $x>0$. Further, because $f(x) \geqslant 0$ for $x \geqslant 0$ with equality if and only if $x=0$, *f* is strictly convex. We apply this observation in order to derive bounds on homozygosity obtained by Rosenberg and Jakobsson (2008), as well as to obtain bounds on $\alpha$-homozygosity. We also rely on the fact that the Shannon–Weaver index is strictly Schur-concave as a consequence of Corollary 2.4 together with the fact that $f(x)=-x\log x$ is strictly concave for $x \geqslant 0$.

We now derive bounds on homozygosity, $\alpha$-homozygosity, the Shannon–Weaver index, and the Rényi entropy that quantify the constraints placed by *M*. We will see that the majorization approach not only reproduces the results of Rosenberg and Jakobsson (2008) and Reddy and Rosenberg (2012), it also produces bounds for the more general $\alpha$-homozygosity.

### Unspecified number of distinct alleles

The following result was obtained by Rosenberg and Jakobsson (2008). This result indicates that for a fixed value of the frequency of the most frequent allele, homozygosity is maximized by setting as many allele frequencies as possible equal to the largest frequency, with at most one nonzero allele frequency remaining.

#### Theorem 3.1

(Theorem 2, Rosenberg and Jakobsson 2008) Consider a sequence of the allele frequencies at a locus, $(p_i)_{i=1}^\infty$, with $p_i\in [0,1)$, $\sum _{i=1}^\infty p_i=1$, $J=\sum _{i=1}^\infty p_i^2$, $M=p_1$, and $i<j$ implies $p_i\geqslant p_j$. Then

(i) $$J>M^2$$, and
(ii) $$J\leqslant 1-M(\lceil M^{-1}\rceil -1)(2-\lceil M^{-1}\rceil M)$$,

with equality if and only if $p_i=M$ for $1\leqslant i\leqslant K-1$, $p_K=1-(K-1)M$, and $p_i=0$ for $i>K$, where $K=\lceil J^{-1}\rceil =\lceil M^{-1}\rceil$.

This result was the main mathematical result of Rosenberg and Jakobsson (2008); to obtain it in a manner that provides a broader mathematical perspective, we prove a general theorem, Theorem 3.2, which applies to a general class of functions that includes homozygosity. By checking that the theorem applies to *J*, Theorem 3.1 will follow as a corollary.

Let $\Delta =\bigcup _{I=1}^\infty \Delta _{I-1}$ be the set of all nonnegative vectors of finite length summing to 1. For a real number *x*, we use $\{x\}=x-\lfloor x\rfloor$ to denote its fractional part.

#### Theorem 3.2

Let $F:\Delta \rightarrow \mathbb {R}$ be given by $F(\mathbf {p})=\sum _{i=1}^\infty f(p_i)$, where $f:[0,\infty )\rightarrow [0,\infty )$, $f(0)=0$, and *f* is continuous and convex. Assume that $F(\mathbf {p})$ is bounded for all $\mathbf {p}\in \Delta$. Suppose the number of nonzero entries in $\mathbf {p}$ is at most *I*, $p_1=M\in (0,1)$, and $p_i\geqslant p_j$ whenever $i<j$. Define $K=\lceil M^{-1}\rceil$. Then for all $I\geqslant K$,

(i) $$F \geqslant f(M)$$, and
(ii) $$F\leqslant \lfloor M^{-1}\rfloor f(M)+f(\{M^{-1}\}M)$$.

Equality of *F* with the upper bound in (ii) occurs if $p_i=M$ for $1\leqslant i\leqslant K-1,~p_{K}=1-(K-1)M$, and $p_i=0$ for $i>K$. If *f* is strictly convex, then this is the only configuration at which equality holds. If *f* is strictly convex, then the inequality in (i) is strict.

Theorem 3.2 indicates that any bounded function *F* that can be expressed as a sum of convex functions *f* of each argument and that satisfies other mild conditions is bounded below by *f*(*M*), where *M* is the largest component of the vector $\mathbf {p}$ at which *F* is being evaluated, and it cannot exceed the value given by evaluating *F* at the vector $\overset{\sim }{\mathbf {p}}=(M,\ldots ,M,\{M^{-1}\}M,0,\ldots )\in \Delta$, where $\overset{\sim }{\mathbf {p}}$ includes $\lfloor M^{-1} \rfloor$ components of size *M*.

To see how Theorem 3.1 follows from the more general Theorem 3.2, set $f(x)=x^2$. We know that *f* is continuous and strictly convex, with nonnegative range. It satisfies $f(0)=0$, and moreover *F* is bounded, as $0\leqslant F(\mathbf {p})=\sum _{i=1}^\infty p_i^2\leqslant \sum _{i=1}^\infty p_i=1$. The expressions in Theorem 3.1i, ii follow after inserting $f(x)=x^2$ into Theorem 3.2. Indeed,

$$\begin{aligned} \lfloor M^{-1}\rfloor f(M)+f(\{M^{-1}\} M)= & {} \lfloor M^{-1}\rfloor M^2 + [(M^{-1}-\lfloor M^{-1}\rfloor )M]^2 \\= & {} 1 - M(\lceil M^{-1}\rceil -1)(2-\lceil M^{-1}\rceil M). \end{aligned}$$

To prove Theorem 3.2, we require a lemma concerning convex functions. This straightforward lemma states that for a convex function *f*, if certain conditions are satisfied, then the sum $\sum _{i=1}^\infty f(x_i)$ does not exceed $f\left( \sum _{i=1}^\infty x_i\right)$.

#### Lemma 3.3

Let $f:[0,\infty )\rightarrow [0,\infty )$ be a continuous function with $f(0)=0$. Consider $(x_i)_{i=1}^\infty$, with each $x_i \in [0,\infty )$ and with $\sum _{i=1}^\infty x_i=s<\infty$ and $\sum _{i=1}^\infty f(x_i)<\infty$. If *f* is convex, then

$$\begin{aligned} f\left( \sum _{i=1}^\infty x_i\right) \geqslant \sum _{i=1}^\infty f(x_i). \end{aligned}$$

#### Proof

The proof relies on Karamata’s inequality applied to sequences of increasing length. First, observe that for any positive integer *n*, $(x_1+\cdots +x_n,0,\ldots ,0)\succ (x_1,x_2,\ldots ,x_n)$. Hence, by Karamata’s inequality applied to the convex function *f* (Theorem 2.5),

$$\begin{aligned} f\left( \sum _{i=1}^n x_i\right) = f\left( \sum _{i=1}^n x_i\right) + (n-1)f(0) \geqslant \sum _{i=1}^nf(x_i). \end{aligned}$$

Consider the limit as $n\rightarrow \infty$. As a bounded increasing sequence, $\sum _{i=1}^nx_i$ converges to its limit, $\lim _{n\rightarrow \infty }\sum _{i=1}^nx_i=s$. Because *f* is continuous, $f(s)=\lim _{n\rightarrow \infty }f(\sum _{i=1}^nx_i)$. Moreover, $\lim _{n\rightarrow \infty } \sum _{i=1}^nf(x_i)=\sum _{i=1}^\infty f(x_i)$, because the sequence of partial sums $\sum _{i=1}^nf(x_i)$ is a monotone increasing sequence that is bounded. Compiling these results, we have

$$\begin{aligned} f(s)=\lim _{n\rightarrow \infty }f\left( \sum _{i=1}^nx_i\right) \geqslant \lim _{n\rightarrow \infty } \sum _{i=1}^nf(x_i)=\sum _{i=1}^\infty f(x_i). \qquad \qquad \qquad \qquad \qquad \qquad \Box \end{aligned}$$

#### Proof of Theorem 3.2

(ii) Choose *M* with $0< M < 1$. We must show that for any $\mathbf {p}\in \Delta$ with $p_1=\max (p_i)_{i=1}^\infty =M$, $F(\mathbf {p})\leqslant F(\overset{\sim }{\mathbf {p}})$, where $\overset{\sim }{\mathbf {p}}=(M,\ldots ,M,\{M^{-1}\}M,0,\ldots )$. Let $\mathbf {p}=(p_i)_{i=1}^\infty$ be any vector in $\Delta$ with $p_1=M$. Function *f* is assumed to be nonnegative, continuous, and convex with $f(0)=0$. Because $\mathbf {p}$ is in $\Delta$, $\sum _{i=1}^\infty p_i=1$. Because $\sum _{i=1}^\infty f(p_i)=F(\mathbf {p})$ and $F(\mathbf {p})$ is bounded, *f* satisfies the conditions of Lemma 3.3. Hence, for each *n*,

$$\begin{aligned} f\left( \sum _{i=n}^\infty p_i\right) \geqslant \sum _{i=n}^\infty f(p_i). \end{aligned}$$

Let *N* be the minimal value of *n* such that $\sum _{i=n}^\infty p_i\leqslant M$. Let $\sum _{i=N}^\infty p_i=s$. Then

3.4 $$\begin{aligned} \left( \sum _{i=1}^{N-1} f(p_i) \right) +f(s)= & {} \left( \sum _{i=1}^{N-1} f(p_i) \right) +f\left( \sum _{i=N}^\infty p_i\right) \nonumber \\\geqslant & {} \sum _{i=1}^{N-1} f(p_i)+\sum _{i=N}^\infty f(p_i). \end{aligned}$$

We now claim that the left-hand side of Eq. 3.4 is bounded above by $\lfloor M^{-1}\rfloor f(M)+f(\{M^{-1}\}M)$, which is what we wish to show.

Let $\mathbf {t}=(M,\ldots ,M, \{M^{-1}\}M)$, where the first $\lfloor M^{-1}\rfloor$ terms all equal *M*; $\mathbf {t}$ is equal to $\overset{\sim }{\mathbf {p}}$, except that $\overset{\sim }{\mathbf {p}}$ appends infinitely many zeroes. Let $\mathbf {u}=(p_1,\ldots ,p_{N-1},s)$. Because $\mathbf {t}$ and $\mathbf {u}$ possibly have distinct (finite) lengths, define $m=\max (\lfloor M^{-1}\rfloor +1, N)$. Append zeroes to $\mathbf {t}$ or $\mathbf {u}$, so that $\mathbf {t}$ and $\mathbf {u}$ have the same length, *m*. We prove that after this procedure is performed, $\mathbf {t}\succ \mathbf {u}$. This result would immediately imply the claim, because we could then apply Karamata’s inequality (Theorem 2.5) to convex *f* and vectors $\mathbf {t}$ and $\mathbf {u}$ to obtain (ii). Indeed, because $\mathbf {t}\succ \mathbf {u}$ and *f* is convex, denoting $F(x_1,\ldots ,x_m)=\sum _{i=1}^m f(x_i)$, we would have

$$\begin{aligned} \lfloor M^{-1}\rfloor f(M)+f(\{M^{-1}\} M)= & {} \left( \sum _{i=1}^{\lfloor M^{-1}\rfloor } f(M) \right) +f(1-\lfloor M^{-1}\rfloor M) \\= & {} F(\mathbf {t}) \\\geqslant & {} F(\mathbf {u}) \\= & {} \left( \sum _{i=1}^{N-1} f(p_i) \right) +f(s). \end{aligned}$$

Here, we use the fact that $f(0)=0$, so that the additional zeroes do not affect $F(\mathbf {t})$ or $F(\mathbf {u})$.

We now verify that $\mathbf {t}\succ \mathbf {u}$. Observe that $\mathbf {t}$ and $\mathbf {u}$ have the same sum. Moreover, because $p_i\leqslant M$ for each $i\leqslant N-1$, it follows that the sum of the *j* largest components of $\mathbf {u}$, where $j\leqslant \lfloor M^{-1}\rfloor$, is bounded above by *jM*. For any $j>\lfloor M^{-1}\rfloor$, the sum of the *j* largest components of $\mathbf {t}$ is 1, which is always an upper bound for the sum of the corresponding components of $\mathbf {u}$. Thus, $\mathbf {t}\succ \mathbf {u}$ as claimed, and (ii) holds.

For the equality condition, note that equality in (ii) requires $F(\mathbf {u})=F(\mathbf {t})$. For strictly convex *f*, $F(\mathbf {u})=F(\mathbf {t})$ requires that $\mathbf {u}$ be a permutation of $\mathbf {t}$, as otherwise, we would have $F(\mathbf {t}) > F(\mathbf {u})$ by the strict Schur-convexity of *F* that follows from Corollary 2.4. Because the components of $\mathbf {u}$ and $\mathbf {t}$ are arranged in decreasing order, equality in (ii) requires that $\mathbf {u}=\mathbf {t}$. Hence, vectors $\overset{\sim }{\mathbf {p}}$ are the only vectors in $\Delta$ that achieve equality in (ii).

(i) Observe that for any $\mathbf {p}\in \Delta$ with $p_1=M$, we have, by the nonnegativity of *f* on $[0,\infty )$,

$$\begin{aligned} F(\mathbf {p})=\sum _{i=1}^\infty f(p_i)\geqslant f(p_1)=f(M). \end{aligned}$$

In the case that *f* is strictly convex and $p_1<1$, $F(\mathbf {p})=\sum _{i=1}^\infty f(p_i)\geqslant f(p_1)+f(p_2)$. For $p_1<1$ and $\mathbf {p}\in \Delta$, $p_2>0$. Because *f* is strictly convex, $f(0)=0$, and $p_2>0$, $f(p_2)>0$. Hence $F(\mathbf {p})>f(p_1)$, and the inequality in (i) is strict. $\square$

Note that for convenience, in the statement of Theorem 3.2, both $\lfloor M^{-1} \rfloor$ and $\lceil M^{-1} \rceil$ appear. In verifying (ii), we have simultaneously considered two cases for which the proof proceeds in the same way: if *M* is the reciprocal of an integer, then $\lfloor M^{-1} \rfloor = \lceil M^{-1} \rceil$, and otherwise, $\lfloor M^{-1} \rfloor = \lceil M^{-1} \rceil - 1$. In the case that *M* is the reciprocal of an integer, $\mathbf {t}$ has a zero in position $\lfloor M^{-1} \rfloor + 1$ and $\{ M^{-1} \} = 0$. The proof is not affected by these values of 0.

Having proven Theorem 3.2 and having shown that it implies Theorem 3.1, we now proceed to show that it implies a similar result for the more general $\alpha$-homozygosity. Recall that $\alpha$-homozygosity $J^\alpha (\mathbf {p})$ is defined by raising each component of an allele frequency vector $\mathbf {p}$ to the $\alpha$th power and taking the sum across components. Because $f(x)=x^\alpha$ is convex, $J^\alpha$ is Schur-convex. Further, $J^\alpha (\mathbf {p})=\sum _{i=1}^\infty f(p_i)$ is continuous and nonnegative on $\Delta$ and satisfies $f(0)=0$. We also see that $J^\alpha (\mathbf {p})\leqslant \sum _{i=1}^\infty p_i=1$ on $\Delta$ and is hence bounded, as $p_i^\alpha \leqslant p_i$ for $0 \leqslant p_i \leqslant 1$ and $\alpha > 1$, so that $\sum _{i=1}^\infty p_i^\alpha \leqslant \sum _{i=1}^\infty p_i = 1$. Consequently, Theorem 3.2 provides bounds on the values of $J^\alpha$ for a fixed value $p_1=M$ of the largest allele frequency.

#### Corollary 3.5

(Bounds for $\alpha$-homozygosity) Suppose $\mathbf {p}\in \Delta$, $p_1=M$ is fixed with $M \in (0,1]$, and $p_i\geqslant p_j$ whenever $i<j$. Then

$$\begin{aligned} M^\alpha \leqslant J^\alpha (\mathbf {p})\leqslant \lfloor M^{-1}\rfloor M^\alpha +\left( \{M^{-1}\}M\right) ^\alpha . \end{aligned}$$

Equality with the upper bound occurs if and only if $\mathbf {p}=(M,\ldots ,M,1-\lfloor M^{-1}\rfloor M, 0, \ldots )$. Equality with the lower bound is achieved if and only if $M=1$.

#### Proof

Both bounds and the equality condition for the upper bound follow directly from Theorem 3.2 applied to the function $J^{\alpha }$. Equality in the lower bound is attained only if $f(p_i)=0$ for all $i\geqslant 2$, so that $p_i=0$ for all $i\geqslant 2$ and $M=1$. $\square$

By setting $\alpha =2$ in Corollary 3.5, we recover Theorem 3.1. Thus, the majorization approach not only proves the result of Rosenberg and Jakobsson (2008), it finds that an analogous result holds for $\alpha$-homozygosity for any $\alpha >1$.

**Fig. 1:**
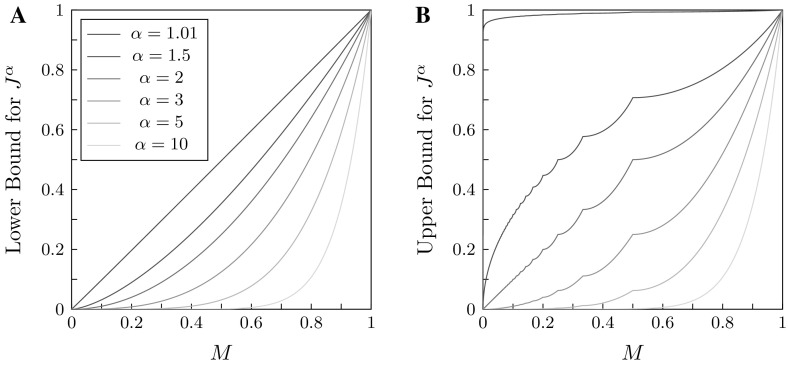
Analytical lower and upper bounds on $\alpha$-homozygosity $J^\alpha$ as functions of the frequency *M* of the most frequent allele. **a** Lower bound and **b** upper bound. The bounds are taken from Corollary 3.5

**Fig. 2:**
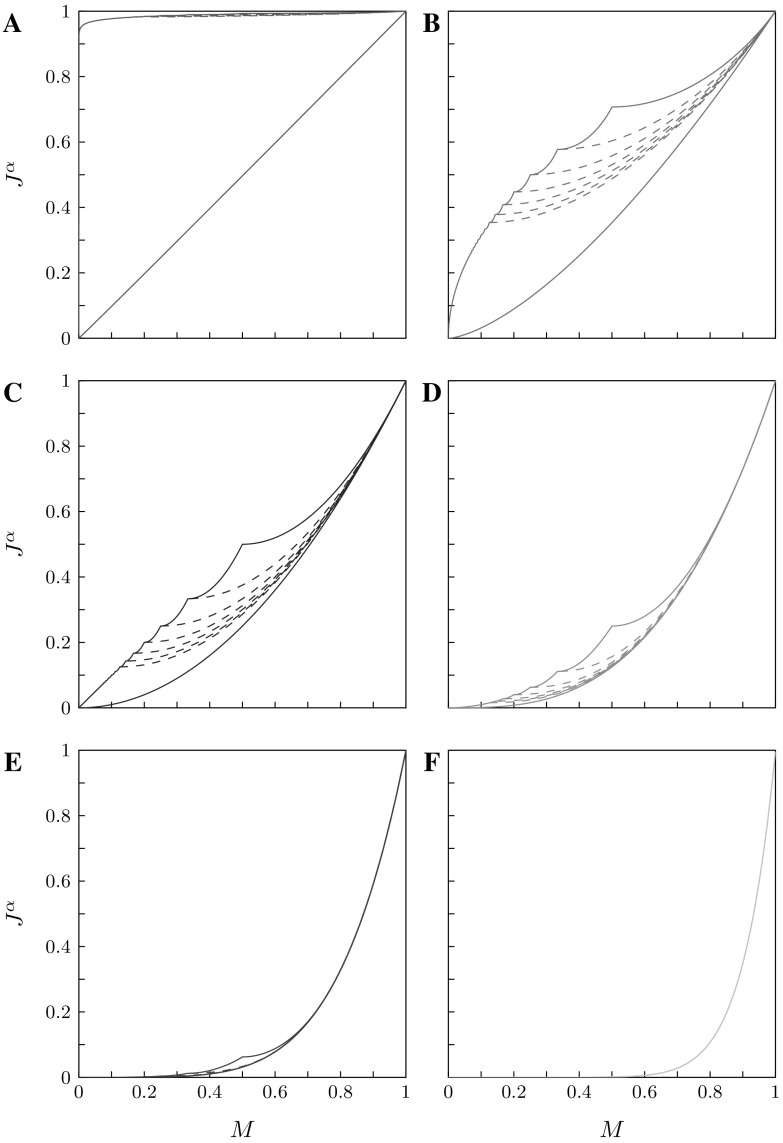
Analytical lower and upper bounds on $\alpha$-homozygosity $J^\alpha$, for different choices of $\alpha$ and the maximal number of distinct alleles *I*. **a** $\alpha =1.01$, **b** $\alpha =1.5$, **c** $\alpha =2$, **d** $\alpha =3$, **e** $\alpha =5$ and **f** $\alpha =10$. The solid lines are the lower and upper bounds obtained from Corollary 3.5. The dotted lines are refinements to the lower bound for different fixed values of *I*, obtained using Corollary 3.13 (from top to bottom, $I=3,4,5,6,7,8$). The upper bound is the same irrespective of the number of distinct alleles, except that it is defined only for $M \geqslant 1/I$. For large $\alpha$, the lower and upper bounds are largely indistinguishable

Figure 1 illustrates the effect of $\alpha$ on the constraints placed by *M* on $\alpha$-homozygosity. Increasing $\alpha$ decreases both the lower bound on $J^\alpha$ (Fig. 1a) and the upper bound (Fig. 1b), shrinking the range of values that $J^\alpha$ can possess (Fig. 2).

This observation can be quantified by noting that the area of the region of permissible values between the lower and upper bounds decreases with increasing $\alpha$. Considering $M \in (0,1]$ and denoting by $L_\alpha$ and $U_\alpha$ the areas of the regions bounded below by the *x*-axis and above by the lower and upper bounds, respectively, the area between the bounds is $S_\alpha =U_\alpha -L_\alpha$. This quantity represents the magnitude of the constraint placed by *M* on $J^\alpha$, measuring both the fraction of the unit square permissible for $(M, J^\alpha )$, and because the interval of permissible values for *M* has size 1, the mean across values of *M* of the interval between the lower and upper bounds. $U_\alpha$ and $L_\alpha$ are computed in the “Appendix”. The area $S_\alpha$ satisfies

3.6 $$\begin{aligned} S_\alpha =\frac{1}{\alpha +1}\sum _{t=1}^\infty \frac{1}{t(t+1)^\alpha }. \end{aligned}$$

If $\alpha =2$, $S_\alpha =(1/3)(1-\sum _{t=2}^\infty 1/t^2)=\frac{2}{3}-\frac{\pi ^2}{18}\approx 0.1184$, as computed by Rosenberg and Jakobsson (2008) for the case of the standard 2-homozygosity. If $\alpha =3$, $S_\alpha =(1/4)(2-\frac{\pi ^2}{6}-\sum _{t=1}^\infty 1/t^3+1)=\frac{3}{4}-\frac{\pi ^2}{24}-\frac{\zeta (3)}{4}\approx 0.03825$, where $\zeta (3)\approx 1.2021$ is Apéry’s constant.

**Fig. 3:**
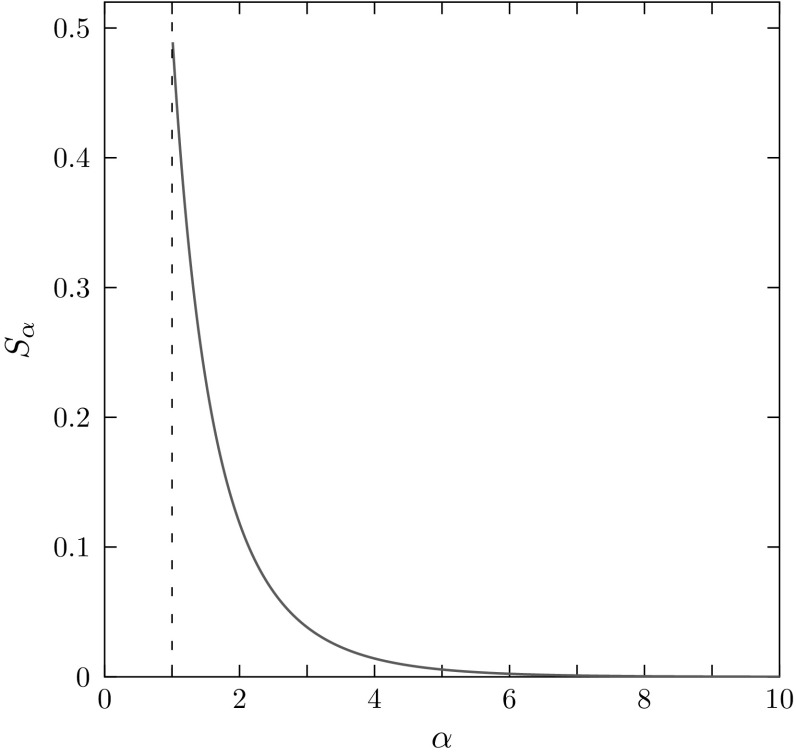
$S_\alpha$, the area of the region of permissible values for $\alpha$-homozygosity, as $\alpha$ increases from 1.01 to 10. $S_\alpha$ is calculated using Eq. 3.6. $S_\alpha$ quickly converges to zero; for example, at $\alpha =10$, $S_\alpha =8.96\times 10^{-5}$

Because $\alpha >1$, the sum $\sum _{t=1}^\infty t^{-(\alpha +1)}$ is a convergent power series, converging to $\zeta (\alpha +1)$, where $\zeta (z)$ denotes the Riemann zeta function. Because $1/[t(t+1)^\alpha ] < 1/t^{\alpha +1}$, we conclude that $S_\alpha \rightarrow 0$ as $\alpha \rightarrow \infty$. Moreover, $S_\alpha$ is monotonically decreasing with increasing $\alpha$. These features can be observed in Fig. 3.

We can also see that as $\alpha$ grows large,

3.7 $$\begin{aligned} \frac{J^\alpha (\mathbf {p})}{M^\alpha }= & {} \sum _{i=1}^\infty \left( \frac{p_i}{M}\right) ^\alpha \nonumber \\= & {} \sum _{\{i:p_i=M\}}1 + \sum _{\{i:p_i< M\}}\left( \frac{p_i}{M}\right) ^\alpha \nonumber \\\approx & {} |\{i:p_i=M]\}|. \end{aligned}$$

Hence, for large $\alpha$, the ratio of $\alpha$-homozygosity to the $\alpha$th power of the frequency of the most frequent allele is approximately the number of alleles that attain the maximal frequency. This result has the consequence that for large powers of $\alpha$, the quotient of $J^\alpha$ and $M^\alpha$ is an approximate indicator of the number of allelic types that achieve the highest frequency.

### Specified number of distinct alleles

So far, we have treated the number of distinct allelic types as unrestricted and possibly infinite. If a finite maximal number of distinct alleles can be specified, however, then the lower bound on homozygosity for a fixed size of the frequency of the most frequent allele can be tightened (Reddy and Rosenberg 2012). We now demonstrate that majorization can recover the lower bound on homozygosity given *M* under the additional constraint of a fixed maximal number of distinct alleles. As was true for an unspecified number of distinct alleles, the approach gives rise to a more general result that enables bound computations for a broader class of similarity and diversity measures.

We extend Theorem 3.2 to produce a result about general convex functions, Theorem 3.9, for the case of a fixed maximal number of distinct alleles. This theorem produces an $\alpha$-homozygosity generalization of the result of Reddy and Rosenberg (2012) on the lower bound on homozygosity at fixed *M*. It furthermore produces bounds on the Shannon–Weaver index and Rényi entropy for a fixed *M* and a fixed maximal number of distinct alleles.

The following result was obtained by Reddy and Rosenberg (2012).

#### Theorem 3.8

(Theorem 2, Reddy and Rosenberg (2012)) Consider a sequence of the allele frequencies at a locus, $(p_1,\ldots ,p_I)$, with $I\geqslant 2$ fixed, such that $p_i\in [0,1)$, $\sum _{i=1}^I p_i=1$, $J=\sum _{i=1}^Ip_i^2$, $M=p_1$, and $i<j$ implies $p_i\geqslant p_j$. Then given $M\in [1/I,1)$,

$$\begin{aligned} \frac{IM^2-2M+1}{I-1}\leqslant J \leqslant 1-M(\lceil M^{-1}\rceil -1)(2-\lceil M^{-1}\rceil M). \end{aligned}$$

Equality in the upper bound occurs under the same conditions as in Theorem 3.1. Equality in the lower bound occurs if and only if $p_i=(1-M)/(I-1)$ for $2 \leqslant i \leqslant I$.

To obtain Theorem 3.8, we prove a more general theorem, Theorem 3.9, which can be viewed as an extension of Theorem 3.2.

#### Theorem 3.9

Let $I\geqslant 2$ be a fixed natural number. Let $F:\Delta _{I-1}\rightarrow \mathbb {R}$ be given by $F(\mathbf {p})=\sum _{i=1}^If(p_i)$, where $f:[0,\infty )\rightarrow \mathbb {R}$ takes on non-negative values on [0, 1], $f(0)=0$, and *f* is continuous and convex. Suppose $p_1=M$ is fixed with $1/I \leqslant M \leqslant 1$, and that $p_i\geqslant p_j$ whenever $i<j$. Then

3.10 $$\begin{aligned} f(M)+(I-1)f\left( \frac{1-M}{I-1}\right) \leqslant F(\mathbf {p})\leqslant \lfloor M^{-1}\rfloor f(M)+f(\{M^{-1}\} M). \end{aligned}$$

Equality in the upper bound occurs under the same conditions as in Theorem 3.2. Equality in the lower bound occurs if $p_i=(1-M)/(I-1)$ for $2 \leqslant i \leqslant I$, and if *f* is strictly convex, then it is achieved only at this configuration. Moreover, if all other conditions on *f* hold but *f* is concave, then the inequalities in Eq. 3.10 are reversed.

#### Proof

Let $\mathcal {D}=\{(p_i)_{i=1}^I \in \Delta _{I-1}:p_1=M,p_i\geqslant p_j\text { whenever } i<j\}$ denote the set of non-increasing vectors in $\Delta _{I-1}$ with first component fixed at *M*.

Consider vectors $\mathbf {t}$ and $\mathbf {u}$, where

3.11 $$\begin{aligned} \mathbf {t}=(M,\ldots ,M, 1-\lfloor M^{-1}\rfloor M,0,\ldots ,0) \end{aligned}$$

has $\lfloor M^{-1}\rfloor$ terms *M* and $I-\lfloor M^{-1}\rfloor - 1$ zeros, and

3.12 $$\begin{aligned} \mathbf {u}=\left( M,\frac{1-M}{I-1},\ldots ,\frac{1-M}{I-1}\right) \end{aligned}$$

has $I-1$ terms $(1-M)/(I-1)$. Observe that $\mathbf {t}$ and $\mathbf {u}$ both lie in $\mathcal {D}$. We must show that for any vector $\mathbf {x}\in \mathcal {D}$, $F(\mathbf {x})$ never exceeds $F(\mathbf {t})$ and never takes a value smaller than $F(\mathbf {u})$.

Because *f* is convex, by Corollary 2.4, *F* is Schur-convex. By Karamata’s inequality (Theorem 2.5), if we can show that $\mathbf {t}$ majorizes every other vector $\mathbf {x}$ in $\mathcal {D}$ and $\mathbf {u}$ is majorized by every other vector $\mathbf {x}$ in $\mathcal {D}$, then the inequalities in Eq. 3.10 follow as a consequence.

We first prove that $\mathbf {t}\succ \mathbf {x}$ for any $\mathbf {x}\in \mathcal {D}$. Observe that for any $\mathbf {x}\in \mathcal {D}$, $x_1,x_2,\ldots ,x_I\leqslant M$, and moreover, $\sum _{i=1}^I x_i=1$. Hence, for each *i* from $1\leqslant i\leqslant \lfloor M^{-1}\rfloor$, the sum of the first *i* terms of $\mathbf {x}$ satisfies $x_1+\cdots +x_i\leqslant iM$, where the right-hand side of the inequality gives the sum of the *i* largest components of $\mathbf {t}$. For $\lfloor M^{-1}\rfloor +1\leqslant i\leqslant I$, observe that $x_1+\cdots +x_i\leqslant 1$, the right-hand side of which is again the sum of the *i* largest components of $\mathbf {t}$. Because the partial sums of $\mathbf {t}$ are at least as large as the partial sums of $\mathbf {x}$ for all *i*, $\mathbf {t}\succ \mathbf {x}$, as claimed.

Next, we prove that $\mathbf {u}\prec \mathbf {x}$ for any $\mathbf {x}\in \mathcal {D}$. Observe that for any $\mathbf {x}$, because its components are arranged in non-increasing order, for $i\geqslant 2$, the sum of the *i* largest components is always at least $M + (i-1)({1-M})/({I-1})$, which corresponds to the sum of the *i* largest components of $\mathbf {u}$. Indeed, if it were not so, then for some *j*, the sum of the *j* largest components of $\mathbf {x}$ would be less than $M+(j-1)({1-M})/({I-1})$. Because the sum of all *I* components of $\mathbf {x}$ is 1, the remaining $I-j$ components would then have sum larger than $(I-j)({1-M})/({I-1})$. This would in turn have as a consequence that at least one of the *j* largest components of $\mathbf {x}$ exceeds $({1-M})/({I-1})$, and one of the $I-j$ smallest components exceeds $({1-M})/({I-1})$, contradicting the non-increasing order of the entries of $\mathbf {x}$. We conclude $\mathbf {u}\prec \mathbf {x}$, as claimed.

If *f* is concave, then *F* is Schur-concave (Corollary 2.4), so the arguments above imply that the inequalities in Eq. 3.10 are reversed. For the equality conditions, note that by the equality condition in Karamata’s inequality (Theorem 2.5), $F(\mathbf {x})=F(\mathbf {t})$ or $F(\mathbf {x})=F(\mathbf {u})$ for strictly convex or strictly concave *f* requires that $\mathbf {x}$ be a permutation of $\mathbf {t}$ or $\mathbf {u}$. It then follows from the nonincreasing order of entries in $\mathbf {x}$ that $\mathbf {x}=\mathbf {t}$ or $\mathbf {x}=\mathbf {u}$. $\square$

#### Proof of Theorem 3.8

We set $f(x)=x^2$ in Theorem 3.9. Observe that for the lower bound expression,

$$\begin{aligned} f(M)+(I-1)f\left( \frac{1-M}{I-1}\right)= & {} M^2+(I-1)\left( \frac{1-M}{I-1}\right) ^2 \\= & {} \frac{IM^2-2M+1}{I-1}. \end{aligned}$$

Because $f(x)=x^2$ is strictly convex, the equality condition in Theorem 3.9 confirms that equality with the lower bound is obtained if and only if $p_2=\cdots = p_I=(1-M)/(I-1)$.

The upper bound expressions for *F* are identical for both Theorems 3.2 and 3.9. The upper bound in Theorem 3.8 and its equality condition both follow from the identity of the upper bounds in Theorems 3.2 and 3.9. $\square$

Recalling that $f(x)=x^\alpha$ is strictly convex for $\alpha > 1$ and making the substitution $f(x)=x^\alpha$, we obtain a more general result for $\alpha$-homozygosities.

#### Corollary 3.13

(Refined bounds for $\alpha$-homozygosity) Suppose $\mathbf {p}\in \Delta _{I-1}$, where $I\geqslant 2$ is fixed, $p_1=M$ is fixed with $M \in [1/I, 1]$, and $p_i\geqslant p_j$ whenever $i<j$. Then

$$\begin{aligned} M^\alpha +\frac{(1-M)^\alpha }{(I-1)^{\alpha -1}}\leqslant J^\alpha (\mathbf {p})\leqslant \lfloor M^{-1}\rfloor M^\alpha +\left( \{M^{-1}\}M\right) ^\alpha . \end{aligned}$$

Equality in the upper bound occurs under the same conditions as in Corollary 3.5. Equality in the lower bound occurs if and only if $p_i=({1-M})/({I-1})$ for $2 \leqslant i \leqslant I$.

Figure 2 shows the lower and upper bounds on $J^\alpha$ for various values of $\alpha$ in both the case of an unspecified number of distinct alleles and in cases with various fixed values of *I*. For each $\alpha$, fixing the maximal number of distinct alleles further constrains the relationship between *M* and $J^\alpha$ compared to the unspecified case; examining the lower bounds in Corollaries 3.13 and 3.5, we see that the lower bound on $\alpha$-homozygosity is enlarged by the second nonzero term $(1-M)^\alpha / (I-1)^{\alpha -1}$ in the case of a specified number of distinct alleles.

We can quantify the additional constraint imposed by fixing the maximal number of distinct alleles by noting that the area of the region of permissible values shrinks from the original value of $S_\alpha =[1/(\alpha +1)]\sum _{t=1}^\infty 1/[t(t+1)^\alpha ]$ (Eq. 3.6). First, the constraint on *I* forces $M\geqslant 1/I$. We denote by $L^I_\alpha$ and $U^I_\alpha$ the areas of the regions of the unit square bounded above by the lower and upper bounds, respectively, and we compute these areas in the “Appendix”. Thus, denoting the area of the region of permissible values by $S^I_\alpha =U^I_\alpha -L^I_\alpha$, we have

3.14 $$\begin{aligned} S^I_\alpha =\frac{1}{\alpha +1}\left[ \sum _{t=1}^{I-1} \frac{1}{t(t+1)^\alpha }-\frac{I-1}{I^\alpha }\right] . \end{aligned}$$

This quantity is less than $S_\alpha$ from Eq. 3.6, which both has more positive terms in its summation and does not subtract from the sum the positive quantity $[(1/(\alpha +1)](I-1)/I^\alpha$.

**Fig. 4:**
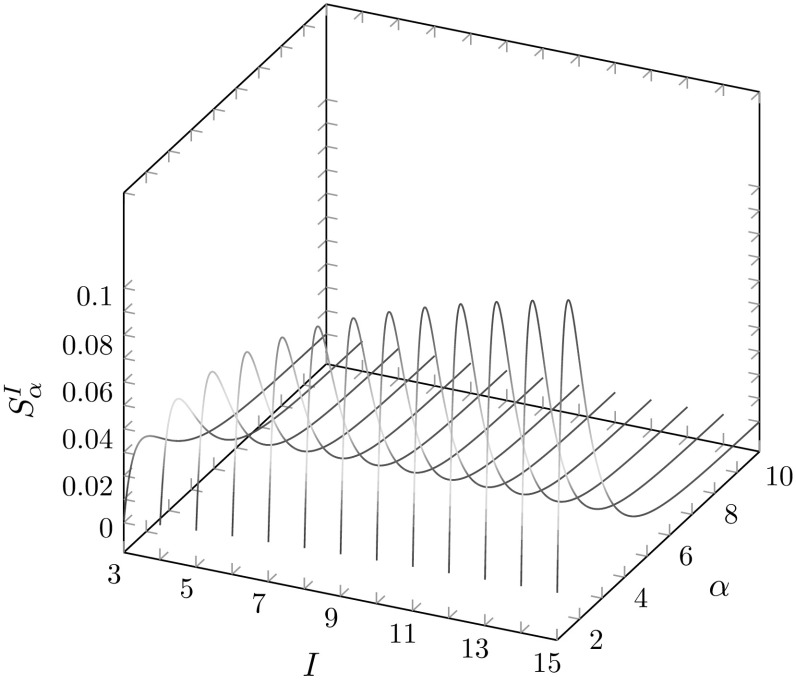
$S_\alpha ^I$, the area of the region of permissible values for $\alpha$-homozygosity, as $\alpha$ increases from 1.01 to 10 and the maximal number of distinct alleles *I* increases from 3 to 15. $S_\alpha ^I$ is calculated using Eq. 3.14. As *I* increases, the curve approaches the shape of the $S_\alpha$ graph in Fig. 3

This result illustrates that if the number of distinct alleles *I* is fixed, then the constraint placed by *M* on $J^\alpha$ is tighter, and depends on both $\alpha$ and *I*. Note that as $I\rightarrow \infty$, $S_\alpha ^I\rightarrow S_\alpha$, as can be seen in Fig. 4. If $\alpha =2$, then we obtain $S^I_\alpha =(I-1)^2/(3I^2)-(1/3)\sum _{t=2}^I 1/t^2$. Dividing this quantity by $1-1/I$, the length of the interval [1 / *I*, 1] of permissible values of *M*, we recover the result of Proposition 7iii of Reddy and Rosenberg (2012) that states that the mean value of the distance between the upper bound and refined lower bound of homozygosity is $1/3-1/(3I)-\{I/[3(I-1)]\}\sum _{t=2}^I 1/t^2$.

Now that we have obtained lower and upper bounds on $\alpha$-homozygosity, we next bound the Shannon–Weaver index *H*. In the case of infinitely many allelic types, the Shannon–Weaver index can be made arbitrarily large even if the frequency $M<1$ of the most frequent allele is specified. Fix *I* and consider the vector $v_I \in \Delta$, defined by

3.15 $$\begin{aligned} \mathbf {v}_I = \left( M,\frac{1-M}{I-1},\ldots ,\frac{1-M}{I-1}\right) , \end{aligned}$$

where $I-1$ entries in the vector have value $(1-M)/(I-1)$. Observe that $H(\mathbf {v}_I)=-M\log M-(1-M)\log [(1-M)/(I-1)]$. As $I\rightarrow \infty$, $H(\mathbf {v}_I)\rightarrow \infty$.

If the number of allelic types is finite, however, then we can apply Theorem 3.9 to obtain bounds on the statistic if the frequency *M* of the most frequent allele is specified. We observed earlier that the Shannon–Weaver index is strictly Schur-concave, and is thus antitone with respect to majorization. In other words, the more majorized a vector is, the smaller the value of *H* it outputs. Using this fact, we have the following bounds on the Shannon–Weaver index for a fixed frequency of the most frequent allele.

#### Corollary 3.16

(Bounds for Shannon–Weaver index) Suppose $\mathbf {p}\in \Delta _{I-1}$, where $I\geqslant 2$ is fixed with $M \in [1/I,1]$, $p_1=M$ is fixed, and $p_i\geqslant p_j$ whenever $i<j$. Then

$$\begin{aligned}&\lfloor M^{-1}\rfloor M\log \frac{1}{M} + (1-M\lfloor M^{-1}\rfloor )\log \left( \frac{1}{1-M\lfloor M^{-1}\rfloor }\right) \leqslant H(\mathbf {p}) \\&\quad \leqslant M\log \frac{1}{M}+(1-M)\log \left( \frac{I-1}{1-M}\right) . \end{aligned}$$

Equality in the upper bound occurs if and only if $p_i=({1-M})/({I-1})$ for $2 \leqslant i \leqslant I$. Equality in the lower bound occurs if and only if $p_i=M$ for $1 \leqslant i \leqslant K-1$, $p_{K}=1-(K - 1)M$, and $p_i=0$ for $i>K$, where $K = \lceil M^{-1} \rceil$.

Here, we adopt the convention that $0\log \infty = -0\log 0 =0$, so that if *M* is the reciprocal of a positive integer, the lower bound reduces to $\log (1/M)$.

#### Proof

We apply Theorem 3.9 to the continuous and concave function $f(x)=-x\log x=x\log (1/x)$, which is both non-negative on [0, 1] and satisfies $f(0)=0$. Reversing the inequalities in Theorem 3.9 owing to the concavity of *f*, for the lower bound, recalling that $\{M^{-1}\}M=1-M\lfloor M^{-1}\rfloor$, we have

$$\begin{aligned}&\lfloor M^{-1}\rfloor f(M)+f(\{M^{-1}\} M) = \lfloor M^{-1}\rfloor M\log \frac{1}{M}\\&\quad + (1-M\lfloor M^{-1}\rfloor )\log \left( \frac{1}{1-M\lfloor M^{-1}\rfloor }\right) . \end{aligned}$$

For the upper bound,

$$\begin{aligned} f(M)+(I-1)f\left( \frac{1-M}{I-1}\right) = M\log \frac{1}{M} + (1-M)\log \left( \frac{I-1}{1-M}\right) . \end{aligned}$$

Because *f* is strictly concave, equality with the upper bound is obtained if and only if $p_2=\cdots = p_I=({1-M})/({I-1})$, and equality with the lower bound is obtained if and only if $p_i=M$ for $1\leqslant i\leqslant K-1$ and $p_{K}=1-(K-1)M$, as implied by Theorem 3.9. $\square$

We can also obtain a more general result for the Rényi entropy $H^\alpha$ for $\alpha \in (0,1) \cup (1, \infty )$. Recall the set $\mathcal {D}$ and vectors $\mathbf {t}$ and $\mathbf {u}$ from the proof of Theorem 3.9, in which it was demonstrated that $\mathbf {t}\succ \mathbf {x}\succ \mathbf {u}$ for any $\mathbf {x}\in \mathcal {D}$.

The Rényi entropies, though not generally concave for $\alpha > 1$, are strictly Schur-concave for all $\alpha > 0$ as a consequence of possessing the weaker property of quasiconcavity (Ho and Verdú 2015). Symmetric, quasiconcave functions are Schur-concave (Marshall et al. 2010, p. 98), and the Schur–Ostrowski criterion (Theorem 2.3) verifies that the Schur-concavity is strict: $(p_i-p_j)(\partial H^\alpha / \partial p_i - \partial H^\alpha / \partial p_j) = [\alpha /(1-\alpha )](p_i-p_j)(p_i^{\alpha -1}-p_j^{\alpha -1})/(p_1^\alpha +\cdots +p_I^\alpha ) \leqslant 0$ with equality if and only if $p_i=p_j$.

It follows from the definition of Schur-concavity that the strictly Schur-concave $H^\alpha$ satisfies $H^\alpha (\mathbf {t})\leqslant H^\alpha (\mathbf {x})\leqslant H^\alpha (\mathbf {u})$, with equality at the lower and upper bounds if and only if $\mathbf {x}=\mathbf {t}$ and $\mathbf {x}=\mathbf {u}$, respectively. The Rényi entropy $H^\alpha$ then has for its lower and upper bounds the quantities $H^\alpha (\mathbf {t})$ and $H^\alpha (\mathbf {u})$, respectively:

3.17 $$\begin{aligned} H^\alpha (\mathbf {t})= & {} \frac{1}{1-\alpha }\log [ \lfloor M^{-1}\rfloor M^\alpha + (1-M\lfloor M^{-1}\rfloor )^\alpha ]. \end{aligned}$$

3.18 $$\begin{aligned} H^\alpha (\mathbf {u})= & {} \frac{1}{1-\alpha }\log \bigg [M^\alpha + (I-1)\left( \frac{1-M}{I-1}\right) ^\alpha \bigg ]. \end{aligned}$$

Considering the Shannon–Weaver index to be the $\alpha =1$ case of the Rényi entropy, we can combine these quantities with Corollary 3.16 to state the following corollary.

#### Corollary 3.19

(Bounds for Rényi entropy) Suppose $\mathbf {p}\in \Delta _{I-1}$, where $I\geqslant 2$ is fixed with $M \in [1/I,1]$, $p_1=M$ is fixed, and $p_i\geqslant p_j$ whenever $i<j$. For $\alpha > 0$,

$$\begin{aligned} H^\alpha (\mathbf {t}) \leqslant H^\alpha (\mathbf {p}) \leqslant H^\alpha (\mathbf {u}), \end{aligned}$$

where $\mathbf {t}$, $\mathbf {u}$, $H^\alpha (\mathbf {t})$, and $H^\alpha (\mathbf {u})$ follow Eqs. 3.11, 3.12, 3.17, and 3.18, respectively, and where $H^1(\mathbf {t})$, $H^1(\mathbf {p})$, and $H^1(\mathbf {u})$ are interpreted as limits as $\alpha \rightarrow 1$. Equality in the upper bound occurs if and only if $p_i=({1-M})/({I-1})$ for $2 \leqslant i \leqslant I$. Equality in the lower bound occurs if and only if $p_i=M$ for $1 \leqslant i \leqslant K-1$, $p_{K}=1-(K - 1)M$, and $p_i=0$ for $i>K$, where $K = \lceil M^{-1} \rceil$.

**Fig. 5:**
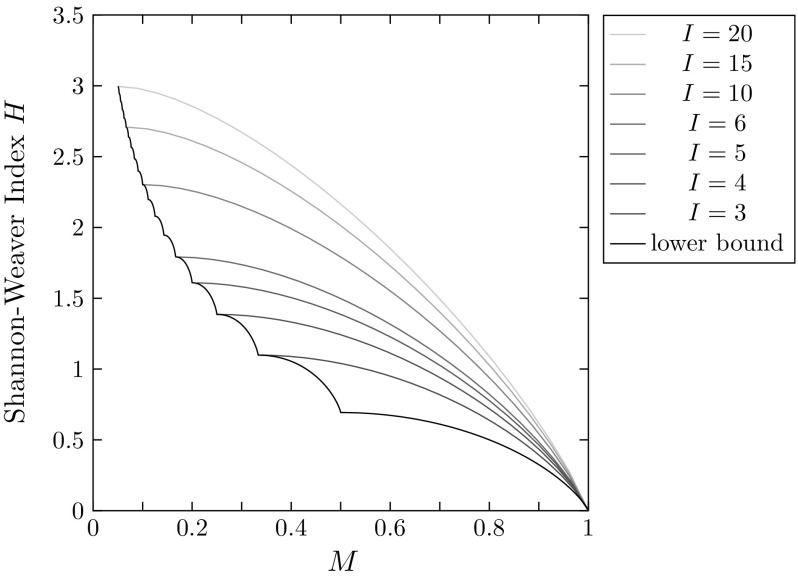
Analytical lower and upper bounds on the Shannon–Weaver index *H* as functions of the frequency *M* of the most frequent allele, for different choices of the maximal number of distinct alleles *I*. According to Corollary 3.16, the lower bound, shown by the solid black curve, does not depend on *I*, except that it is defined only for $M \geqslant 1/I$. As *I* increases, the upper bound—depicted by the blue curves—increases without bound. The increase is slower for large values of *M*

Figure 5 plots the bounds on *H* as a function of *M* for several choices of *I*, illustrating the constraints placed on *H* by both *M* and the number of distinct alleles *I*. As discussed above, *H* is unbounded if the length of the allele frequency vector is unspecified. By fixing the length of the frequency vector, we are able to obtain a finite bound on *H*; for small values of *I*, *H* is tightly constrained as a function of *M*.

Following the approach we adopted for $\alpha$-homozygosity, we can quantify the constraint on *H* as a function of *M* by measuring the area of the region of permissible values of *H*. Given *I*, the upper bound on *H* cannot exceed $\log I$, irrespective of the value of *M* (Legendre and Legendre 1998, pp. 239–245). We denote by $L^I_{SW}$ and $U^I_{SW}$ the areas of the regions of the rectangle $\mathcal {R}=[1/I,1]\times [0,\log I]$ that are bounded above by the lower and upper bounds of *H* given *M*, respectively. We compute these quantities in the “Appendix”, and obtain that the area of the permissible region for *H*, denoted by $S^I_{SW}=U^I_{SW} - L^I_{SW}$, satisfies

3.20 $$\begin{aligned} S^I_{SW}= & {} \frac{(I-1)\log I}{2I} -\frac{1}{2} \sum _{t=1}^{I-1}\frac{\log (t+1)}{t(t+1)}. \end{aligned}$$

The sum in Eq. 3.20 converges as $I\rightarrow \infty$: because $\log t < \sqrt{t}$ for $t \geqslant 1$, $\log (t+1)/[t(t+1)]< 1/(t \sqrt{t+1}) < t^{-3/2}$, so that the sum is bounded above by the convergent sum $\sum _{t=1}^\infty t^{-3/2}$.

Dividing $S^I_{SW}$ by the total area of rectangle $\mathcal {R}$, or $(1-1/I)\log I$, we see that as $I\rightarrow \infty$,

3.21 $$\begin{aligned} \frac{S^I_{SW}}{\left( 1-\frac{1}{I}\right) \log I} = \frac{1}{2} - \frac{I}{2(I-1)\log I} \sum _{t=1}^{I-1}\frac{\log (t+1)}{t(t+1)} \longrightarrow \frac{1}{2}. \end{aligned}$$

Hence, with a large number of allelic types, the permissible values of *H* span about half the area of the rectangle $\mathcal {R}$ in which (*M*, *H*) must lie. Because a positive term is subtracted from $\frac{1}{2}$ in the ratio $S^I_{SW}/[\left( 1-\frac{1}{I}\right) \log I ]$, $S^I_{SW}$ is strictly smaller than $(1/2)(1-1/I)\log I$.

**Fig. 6:**
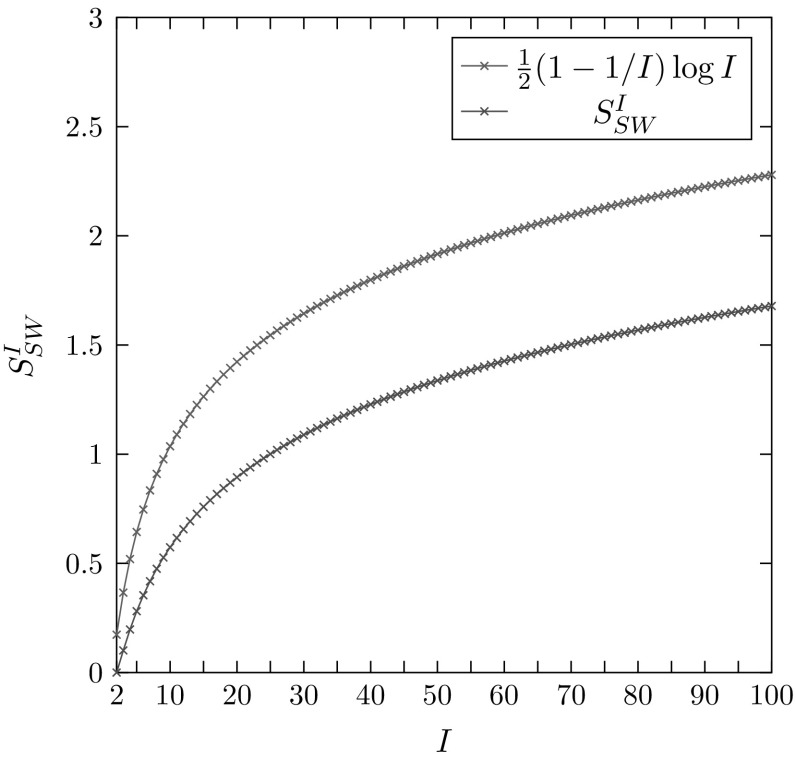
$S^I_{SW}$, the area of the region of permissible values of the Shannon–Weaver index *H* for *I* distinct alleles, alongside the growth of $\frac{1}{2}(1-1/I)\log I$, for values of *I* ranging from 2 to 100. $S^I_{SW}$ is calculated from Eq. 3.20. Note that it is always true that $S^I_{SW}< \frac{1}{2}(1-1/I)\log I$

Figure 6 plots $S^I_{SW}$ as a function of *I*. With the graph of $(1/2)(1-1/I)\log I$ shown for comparison, we observe that $S^I_{SW}$ is bounded above by $(1/2)(1-1/I)\log I$, and hence also by $(1/2)\log I$, in accord with our analytical observation.

## Application to data

To illustrate the bounds on $\alpha$-homozygosity and the Shannon–Weaver index, we plot data on allele frequencies from a human population-genetic data set alongside the bounds in Corollaries 3.5 and 3.16. We use 783 multiallelic microsatellite loci studied in 1048 individuals drawn from populations worldwide (Rosenberg et al. 2005). For each locus, we treat sample frequencies as parametric allele frequencies, and we obtain $J^\alpha$, *H*, and *M* in the full collection of individuals. The missing data rate was 3.7% (Rosenberg et al. 2005), and the minimum nonzero frequency was 1 / 2094, observed at a locus with one missing individual.

**Fig. 7:**
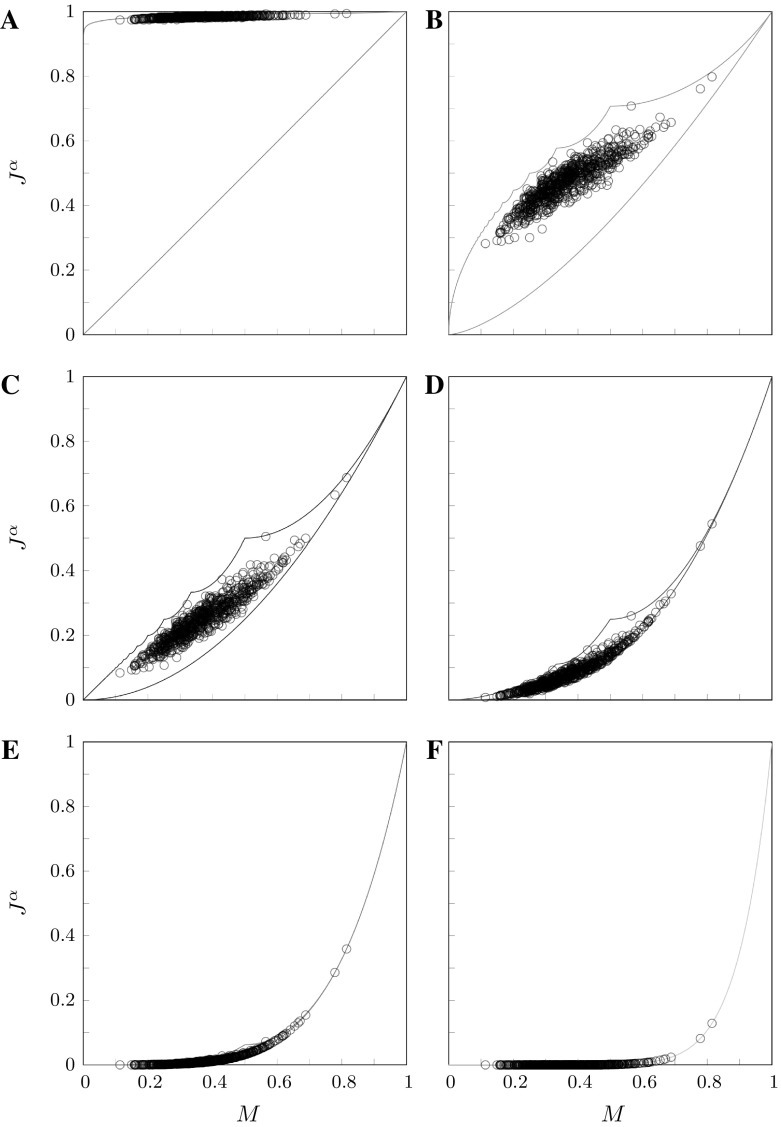
$\alpha$-homozygosities of 783 microsatellite loci. The permissible region for $\alpha$-homozygosity is shown as a function of the frequency *M* of the most frequent allele (Corollary 3.5). **a** $\alpha =1.01$, **b** $\alpha =1.5$, **c** $\alpha =2$, **d** $\alpha =3$, **e** $\alpha =5$ and **f** $\alpha =10$

Figure 7 plots $\alpha$-homozygosity for each of several choices of $\alpha$, considering all 783 loci. For smaller $\alpha$, $\alpha$-homozygosities tend to lie nearer to the upper bound in the permissible range. In fact, for $\alpha =1.01$, $\alpha$-homozygosity is close to the theoretical maximum. Because low values of $\alpha$ give substantial weight to subsequent alleles after the most frequent one, the fact that such alleles tend to have nontrivial frequencies (at least 1 / 2094) causes $\alpha$-homozygosity at low $\alpha$ to lie near the upper bound. As $\alpha$ increases, the $\alpha$-homozygosities shift away from the upper bound. For $\alpha =5$ and $\alpha =10$, the tight constraints placed by *M* on $J^\alpha$ reduce the range of permissible values, and data points are close to the lower bound of a tight range.

In Fig. 7D, the $\alpha$-homozygosity values of three loci stand out for lying close to their corresponding theoretical maxima. The allele frequencies associated with these loci, arranged in decreasing order, appear in Table 1. In each case, $\alpha$-homozygosity lies close to the maximum because for a value of $M>1/2$, the allele frequency vector approximates the scenario with frequencies *M* and $1-M$ that produces the maximal $J^\alpha$.

More precisely, recalling that $\alpha$-homozygosity is strictly Schur-convex for all $\alpha >1$, Corollary 3.5 indicates that $\alpha$-homozygosity is maximized by setting as many allele frequencies as possible equal to the largest frequency, with at most one nonzero allele frequency remaining. Hence, for the values of *M* for the three loci in Table 1, the frequency vectors that maximize $\alpha$-homozygosity are (0.8146, 0.1854, 0, 0, 0, 0, 0, 0), (0.7785, 0.2215, 0, 0, 0, 0, 0) and (0.5646, 0.4354, 0, 0, 0). These vectors are similar to the actual frequency vectors in the table: each locus has a second allele with frequency close to $1-M$, so that the frequency vector approaches the configuration $(M,1-M,0,\ldots ,0)$ that achieves the maximal $J^\alpha$ for $M\geqslant 1/2$.

Interestingly, one of the three loci, TGA012P, had previously been chosen as a particularly clear example of a loss of alleles that occurred during ancient bottlenecks that accompanied human migrations outward from Africa (Figure 2 of Rosenberg and Kang 2015). All six rare alleles at the locus occur in Africa, three of them exclusively so; in Native Americans, distant from the initial source of human genetic diversity in Africa, only the most frequent allele is present. The pattern accords with a scenario in which human migrants out of Africa possessed only a subset of the available alleles, only the most frequent of which was present in later migrants into the Americas. Indeed, because rare alleles are often exclusive to Africa, an allele frequency vector with many rare alleles and with second highest frequency near $1-M$ is a potential candidate for clearly illustrating the loss of alleles during human migrations.

**Table 1:** Three loci whose $\alpha$-homozygosity values lie close to the theoretical maxima associated with their values for the frequency of the most frequent allele

Locus	Allele frequency vector
TGA012P	(0.8146, 0.1520, 0.0203, 0.0048, 0.0029, 0.0029, 0.0015, 0.0010)
TATC059	(0.7785, 0.1604, 0.0394, 0.0126, 0.0061, 0.0025, 0.0005)
D6S2522	(0.5646, 0.4313, 0.0015, 0.0015, 0.0010)

Allele frequencies are listed in descending order

**Fig. 8:**
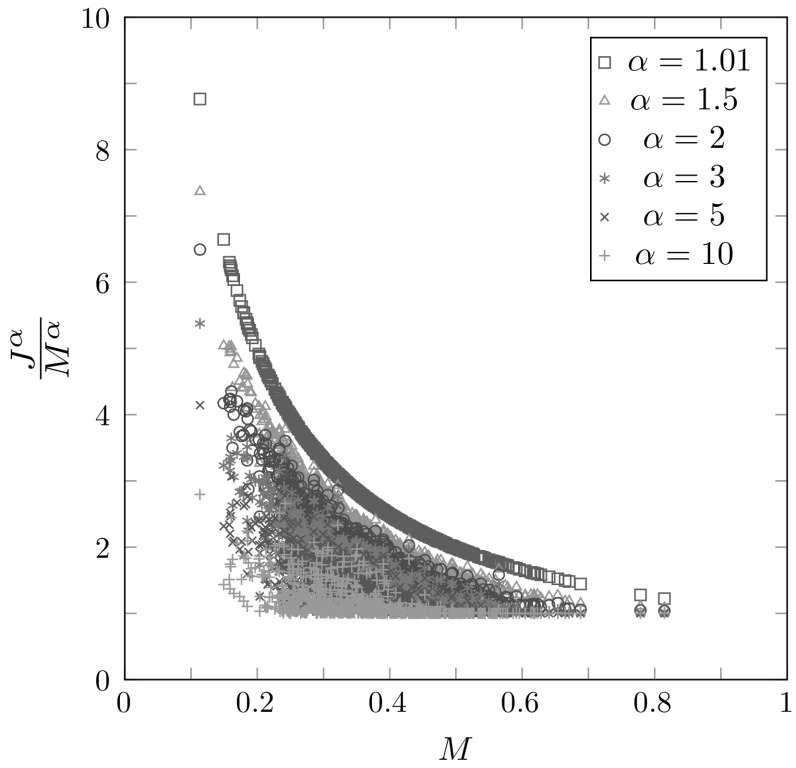
$J^\alpha /M^\alpha$, the ratio of $\alpha$-homozygosity and the frequency *M* of the most frequent allele raised to the power $\alpha$, as a function of *M*, for 783 microsatellite loci. As *M* increases, the values of the ratios for the same locus and different $\alpha$ are closer together

Figure 8 examines the ratio $J^\alpha /M^\alpha$ for the loci for each of several values of $\alpha$. For small *M*, the values of $J^\alpha /M^\alpha$ for a locus vary considerably with $\alpha$, whereas for large *M*, they are similar across $\alpha$ values. Recalling that for large $\alpha$, $J^\alpha /M^\alpha$ approximates the number of distinct alleles with frequency *M*, we can identify from among the larger values of $J^{10}/M^{10}$ the loci with multiple alleles of comparable frequency to *M*. For example, the leftmost locus, with $J^{10}/M^{10} \approx 3$, corresponds to locus GGAT2C03, whose three most frequent alleles have similar frequencies, 0.1136, 0.1091, and 0.1067. Locus GATA88F03P has 0.2404 and 0.2399 for its two highest frequencies, with $J^{10}/M^{10} \approx 2.0108$, and locus D10S1423 has $p_1=0.3069$ and $p_2=0.3064$, with $J^{10}/M^{10} \approx 1.9968$. For most loci, however, the nearest integer to $J^{10}/M^{10}$ is 1, indicating that one allele is substantially more frequent than the others.

**Fig. 9:**
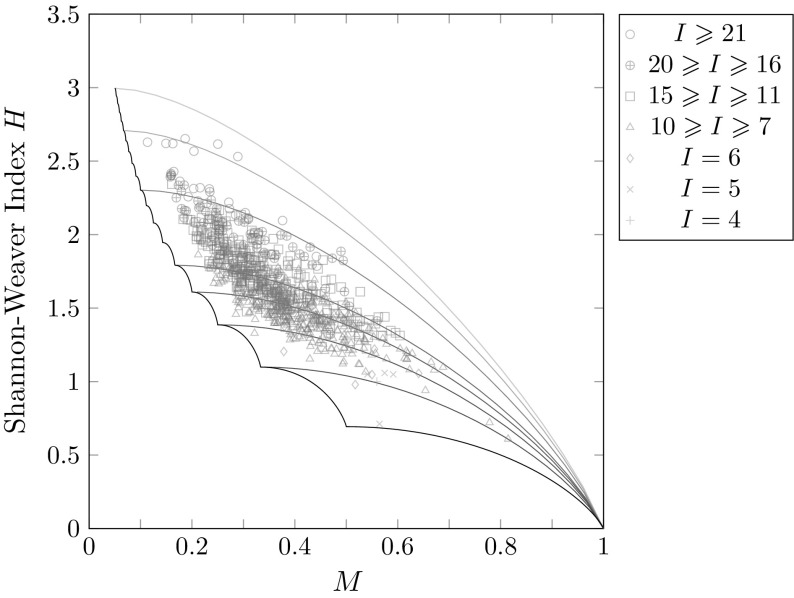
Shannon–Weaver indices of 783 microsatellite loci together with the lower bound of Corollary 3.16 and upper bounds corresponding to different choices of the number *I* of distinct alleles. As in Fig. 5, from bottom to top the upper bound curves correspond to $I=3$, 4, 5, 6, 10, 15, and 20. Each of the 783 microsatellite loci is placed into a bin associated with its number of distinct alleles *I*. The bins correspond to $I=4$ (1 locus), $I=5$ (4 loci), $I=6$ (10 loci), $10\geqslant I\geqslant 7$ (337 loci), $15\geqslant I\geqslant 11$ (311 loci), $20\geqslant I\geqslant 16$ (90 loci), and $I\geqslant 21$ (30 loci). The largest value of *I* across loci is 35

In Fig. 9, we plot the Shannon–Weaver indices of the loci alongside the lower bound and upper bounds for different choices of the number of distinct alleles *I*, in accord with Corollary 3.16. The upper bound lines classify the Shannon–Weaver indices into multiple regions, with a data point lying above an upper bound line only if the number of nonzero allele frequencies at the locus exceeds the number of distinct alleles *I* associated with the line. We find in Fig. 9 that the Shannon–Weaver indices for the 783 loci mostly lie well below the theoretical maxima. The highest Shannon–Weaver index observed is $H \approx 2.6521$, at the locus D22S683, for which $M \approx 0.1867$ and $I=32$; this value is lower than the theoretical upper bound of 3.2743 computed at these values of *M* and *I* from Corollary 3.16.

## Discussion

Using majorization, we have obtained bounds on homozygosity, $\alpha$-homozygosity, the Shannon–Weaver index, and the Rényi entropy in relation to the frequency *M* of the most frequent allele. For homozygosity, majorization recovers the bounds obtained by Rosenberg and Jakobsson (2008) in the case of an unspecified number of distinct alleles (Theorem 3.1) and by Reddy and Rosenberg (2012) for a fixed maximal number of distinct alleles *I* (Theorem 3.8). Moreover, in the fixed-*I* case, $\alpha$-homozygosity for arbitrary $\alpha > 1$, the Shannon–Weaver index, and the Rényi entropy for $\alpha > 0$ achieve their extrema at the same allele frequency vectors that produce the minimal and maximal homozygosity (Corollaries 3.13, 3.16, 3.19).

The results not only simplify the derivation of bounds on homozygosity given *M*, they also illustrate that $\alpha$-homozygosity for $\alpha > 1$ behaves similarly to the standard 2-homozygosity in its dependence on *M*, with an increasing influence for *M* as $\alpha$ increases. Owing in part to the diploidy of many species of interest, in which individuals possess two alleles at a locus, 2-homozygosity—the $\alpha =2$ case of $\alpha$-homozygosity—has been a natural statistic for use in measuring genetic variation. Homozygosity represents both the probability that two alleles drawn at random from a population are identical and the probability that the two alleles of a diploid individual have identical types. In $\alpha$-ploids for $\alpha > 2$, the analogous probability that all $\alpha$ allelic copies in an individual at a locus are identical is $\alpha$-homozygosity. As ploidy increases past 2, the probability that all $\alpha$ alleles are identical is more strongly influenced by *M* than it is for diploids. In the extreme case of large $\alpha$, we found that the ratio $J^\alpha / M^\alpha$ approximates the number of distinct alleles whose frequencies are near *M* (Eq. 3.7).

The varying emphasis on *M* of our $\alpha$-homozygosity statistics is of interest in settings in which 2-homozygosity is currently used. Rosenberg and Jakobsson (2008) commented that tests that identify alleles that positive natural selection has driven rapidly to a high frequency by searching for regions with high haplotype homozygosity use homozygosity as a way of detecting scenarios with a high value of the frequency of the most frequent haplotype. Garud and Rosenberg (2015) developed homozygosity-based tests that search for soft selective sweeps—in which positive selection has inflated the frequencies of multiple haplotypes rather than a single haplotype—by focusing on haplotypes other than the most frequent one. In both cases, $\alpha$-homozygosity for different $\alpha$ could potentially be used: small $\alpha < 2$ in the latter soft-sweep case placing more emphasis on subsequent haplotypes, and large $\alpha > 2$ in the former “hard-sweep” case focusing on the highest-frequency haplotype.

Our results on the Shannon–Weaver index can enable further insight into the statistic in population-genetic settings. Although this statistic has been used less often than homozygosity, it is of interest both for its historical use (e.g. Lewontin 1972), for possible comparisons across data types to areas where it appears more frequently, as well as for use in such settings in their own right. In the ecological context, where *H* measures the diversity of a distribution whose frequencies correspond to species abundances rather than allele frequencies, if the number of species is fixed at *I*, then *H* is bounded above by $\log I$ (Legendre and Legendre 1998, pp. 239–245). In Corollary 3.16, however, we have shown that if the largest frequency in the distribution is also specified, then a further constraint is placed on the theoretical maximum of *H*. The upper bound cannot exceed $\log I$, but it is in fact less than $\log I$ except in the case that $M = 1/I$ and all *I* species have equal frequency. Furthermore, as the number of “species” *I* increases without bound, only at most half the area of the rectangle $\mathcal {R}_I = [1/I,1] \times [0, \log I]$ enclosing potential locations of the pair of quantities (*M*, *H*) contains permissible pairs of values for *M* and *H* (Eq. 3.21). Thus, our analysis finds that averaging over permissible values for *M*, *H* is substantially more constrained on average than might be surmised from knowledge that its maximal value across all *M* is $\log I$.

We have found in our data analysis that values near the bounds are obtained by empirical allele frequencies; the loci generate values of $\alpha$-homozygosity and the Shannon–Weaver index that cover much of the permissible range for these quantities, especially for the more intermediate values of $\alpha$. The bounds assist in the interpretation of the distributions across loci of $(M,J^\alpha )$ and (*M*, *H*), describing their placement within the permissible range; the analysis shows that the bounds are useful for clarifying constraints on data points. Outliers in the plots uncover loci of potential interest, with the ratio $J^\alpha / M^\alpha$ identifying loci with similar frequencies for the two or three most frequent alleles, and the proximity to the upper bound of $J^\alpha$ uncovering an illustration of a serial loss of alleles during human migrations.

Our contributions have utilized majorization and the Schur-convexity and Schur-concavity of the functions used in calculating statistics in population genetics. Each statistic we studied captures the intuitive property that for a fixed sum of frequencies and a fixed maximal number of nonzero components, the least majorized vector, containing only a single nonzero entry, has the lowest value of a diversity index and the highest value of a similarity index, and the most majorized vector, containing a maximal number of equal entries, has the lowest value of a similarity index and the highest value of a diversity index. Indeed, a much stronger result holds, in that these statistics or their additive inverses preserve the majorization order of input vectors, and for fixed *I*, their extrema are achieved at the same frequency vectors.

The connection of majorization to the measures suggests that one aspect of assessing if a new proposed diversity or similarity measure is sensible is an evaluation of whether it or its additive inverse preserves majorization. The homozygosity and Shannon–Weaver statistics have this property, as do the $\alpha$-homozygosities and Rényi entropies. Among statistics that are isotone or antitone with respect to majorization, it could then be evaluated if some are preferable for a particular purpose, as noted above for the potential role of different $\alpha$ for detection of different forms of positive selection. Note that the Rényi entropies for $\alpha \in (0,1) \cup (1, \infty )$ do not have the simpler form $\sum _{i=1}^I f(p_i)$ possessed by $\alpha$-homozygosity and the Shannon–Weaver index; our analysis illustrates that the majorization method applies not only to statistics that take on a form $\sum _{i=1}^I f(p_i)$ that sums analogous quantities computed separately for distinct alleles, but also for a broader class of multivariate Schur-concave functions.

This study both contributes new results and brings methods from another context to the study of well-known population-genetic statistics. We have highlighted that the strong dependence of homozygosity on *M* identified by Rosenberg and Jakobsson (2008) and Reddy and Rosenberg (2012) can be either magnified by considering $\alpha$-homozygosity for $\alpha > 2$ or lessened by using $1< \alpha < 2$, and that the Shannon–Weaver index is constrained in an interval of size well below $\log I$ if *M* is specified. We have also observed examples of these theoretical results in computations with data from human populations. We suggest that the fact that the majorization approach obtains new results on statistics as fundamental as variant forms of homozygosity and the Shannon–Weaver index hints that it might have considerable potential to contribute to mathematical bounds on additional population-genetic statistics.

## Appendix

We report here the integrals that evaluate the sizes of the regions lying under upper and lower bounds.

### Unspecified number of distinct alleles

For the integrals of the upper and lower bounds on $\alpha$-homozygosity, $U_\alpha$ and $L_\alpha$, respectively, we have

$$\begin{aligned} U_\alpha= & {} \int _0^1[\lfloor M^{-1}\rfloor M^\alpha +\left( \{M^{-1}\}M\right) ^\alpha ] \, dM\\= & {} \sum _{t=1}^\infty \int _{\frac{1}{t+1}}^{\frac{1}{t}} [\lfloor M^{-1}\rfloor M^\alpha +\left( \{M^{-1}\}M\right) ^\alpha ] \, dM \\= & {} \sum _{t=1}^\infty \int _{\frac{1}{t+1}}^{\frac{1}{t}} [tM^\alpha + (1-tM)^\alpha ] \, dM \\= & {} \frac{1}{\alpha +1}\left[ 1+\sum _{t=1}^\infty \frac{1}{t(t+1)^\alpha }\right] ,\\ L_\alpha= & {} \int _{0}^1 M^\alpha \, dM = \frac{1}{\alpha +1}. \end{aligned}$$

### Specified number of distinct alleles

For the integrals of the upper and lower bounds on $\alpha$-homozygosity when the number of distinct alleles is at most *I*, $U^I_\alpha$ and $L^I_\alpha$, we have

$$\begin{aligned} U^I_\alpha= & {} \int _{\frac{1}{I}}^1\left[ \lfloor M^{-1}\rfloor M^\alpha +(\{M^{-1}\}M)^\alpha \right] \, dM \\= & {} \sum _{t=1}^{I-1} \int _{\frac{1}{t+1}}^{\frac{1}{t}} [tM^\alpha +(1-tM)^\alpha ] \, dM \\= & {} \frac{1}{\alpha +1}\left[ 1-\frac{1}{I^\alpha }+\sum _{t=1}^{I-1} \frac{1}{t(t+1)^\alpha }\right] ,\\ L^I_\alpha= & {} \int _{\frac{1}{I}}^1 \left[ M^\alpha +\frac{(1-M)^\alpha }{(I-1)^{\alpha -1}} \right] \, dM \\= & {} \frac{1}{\alpha +1}\left( 1+\frac{I-2}{I^\alpha }\right) . \end{aligned}$$

For the integrals of the upper and lower bounds on the Shannon–Weaver index when the number of distinct alleles is at most *I*, $U^I_{SW}$ and $L^I_{SW}$, we have

$$\begin{aligned} U^I_{SW}= & {} \int _{\frac{1}{I}}^1\left[ M\log \frac{1}{M}+(1-M)\log \left( \frac{I-1}{1-M}\right) \right] \, dM \\= & {} \frac{1}{2} + \frac{(I-2)\log I}{2I} - \frac{1}{2I},\\ L^I_{SW}= & {} \int _{\frac{1}{I}}^1\left( \lfloor M^{-1}\rfloor M\log \frac{1}{M}\right) \, dM + \int _{\frac{1}{I}}^1(1-M\lfloor M^{-1}\rfloor )\log \left( \frac{1}{1-M\lfloor M^{-1}\rfloor }\right) \, dM \\= & {} \sum _{t=1}^{I-1} \int _{\frac{1}{t+1}}^{\frac{1}{t}}-tM\log M \, dM + \sum _{t=1}^{I-1} \int _{\frac{1}{t+1}}^{\frac{1}{t}}-(1-Mt)\log (1-Mt) \, dM \\= & {} \left( \sum _{t=1}^{I-1} \frac{t}{2}\left[ \frac{\log t}{t^2}-\frac{\log (t+1)}{(t+1)^2}\right] + \sum _{t=1}^{I-1}\frac{t}{4}\left[ \frac{1}{t^2}-\frac{1}{(t+1)^2}\right] \right) \\&+\,\frac{1}{4} \sum _{t=1}^{I-1} \frac{1+2\log (t+1)}{t(t+1)^2}\\= & {} \frac{1}{2} \sum _{t=1}^{I-1}\frac{\log (t+1)}{t(t+1)} + \frac{1}{2}\left( 1-\frac{1}{I}\right) - \frac{\log I}{2I}. \end{aligned}$$
